# Supplementary material for: Uneven distribution of cobamide biosynthesis and dependence in bacteria predicted by comparative genomics
Source: ISME J. 2018 Nov 14;13(3):789–804. doi: 10.1038/s41396-018-0304-9 (PMC6461909; doi:10.1038/s41396-018-0304-9)
Supplement: Supplementary file 1 — Supplementary Materials, Methods and Figures [file 41396_2018_304_MOESM1_ESM.pdf]

Supplementary Materials and Methods

Bioinformatics

Summary of data set characteristics

Genomes in the JGI/IMGer database are classified as "finished" (5,156 genomes), "permanent draft" (39,645 genomes) or "draft" (1 genome). These genomes had been obtained from isolates (42,190), single cell genomics (1,401), genome-resolved metagenomics (2,095), and other methods (16).

The filtered data set contains 1,621 finished genomes, 9,815 permanent draft genomes, and 0 draft genomes. 10,390 were obtained from isolates, 135 from single cell genomics, 908 from genome-resolved metagenomics, and 3 from other methods.

Creation of *bzaABCDEF* HMMs

Generally, identified proteins were aligned in Jalview, made non-redundant at 90% amino acid identity, and cleaned up (Waterhouse *et al.*, 2009). The final seed alignment was exported and used to create an HMM with the HMMER3 software tool, hmm3build (Eddy, S. R., 2015). These six HMMs were used with the HMMER3 software tool hmm3search to search through fifteen genomes chosen from Hazra *et al*. (2015) to test their functionality and ability to identify the presence of genes from the pathway (Eddy, 2015). In all cases, the genes encoding proteins scoring against the HMMs were found in close proximity to other benzimidazole and cobamide biosynthesis genes, giving confidence to their assigned function.

The three ThiC-like proteins, BzaA, BzaB and BzaF, were analyzed together in order to create models able to distinguish between each of these and other ThiC homologs. Only proteins suspected to be the correct Bza based on scoring against the previous BzaB HMM (TIGR04386) were selected for successive iterations of PSI-BLAST (Altschul *et al.*, 1997) and then the seed alignments for each protein. Training Set Builder (TSB) (Haft and Haft, 2017) was then used to collect ThiC-like proteins, and these homologs were classified according to the newly created BzaA, BzaB, and BzaF HMMs. To refine the new models, we checked the genomic position of the matches, so that *bzaA* and *bzaB* were found within 2000 nucleotides of each other and never in the same genome as *bzaF*, and this analysis was used to determine the trusted cutoff for each of the three HMM profiles.

BzaD and BzaE were examined together due to their similarity. Using known examples of BzaD and BzaE, we used PSI-BLAST to create initial alignments for BzaD and BzaE. Since the set of experimentally categorized sequences was small, the correct amount to bound the PSI-BLAST iterations was not known. We initially chose to use a permissive cutoff, but were able to use the similarity between BzaD and BzaE to provide some bounding conditions. If sequences we captured by both BzaD and BzaE, or if BzaE sequences were hit by the existing BzaD HMM we repeated with less permissive identity thresholds. As with BzaA, BzaB and BzaF, TSB was used to expand out from the initial alignments, adjust the models for accuracy, and assign trusted cutoffs.

To build an initial BzaC HMM, we used the process described above. During evaluation of the HMM cutoff, we discovered that there were short, low-scoring hits to the HMM that co-localized with other genes in the cobamide biosynthesis pathway, forcing us to consider these proteins as a possible related family or as an alternate form of BzaC. However, the lack of homology between these unidentified BzaC-like proteins, the observation of both N and C terminal truncations, and the observation that they were never found in genomes that lacked a

full-length BzaC protein led to the conclusion that these short proteins were degraded and non-functional versions of BzaC and not an additional family. As such, we were then able to validate the cutoffs of the original model.

To download the protein sequences for each genome in our data set, the JGI/IMGer genome entries were matched to GenBank and RefSeq entries (accessed Aug 2, 2017) (Benson *et al.*, 2013; O'Leary *et al.*, 2016). To do so, the GenBank and RefSeq entries were matched by bio sample accession, species name and strain designation, or IMG-taxon ID. Most genomes in the JGI/IMGer dataset had available protein files in these databases (10591 out of 11436).

BLASTP search of putative tetrapyrrole precursor auxotrophs

We queried the genomes of the 201 predicted tetrapyrrole precursor auxotrophs using BLASTP on IMGer using the default settings to only return hits with an E-value less than 1e-5. For the Alphaproteobacterial ALA synthase HemA, we used the protein from *Rhodobacter sphaeroides* with a cutoff of an E-value less than 1e-130 (GenPept C49845). *Clostridium saccharobutylicum* DSM 13864 HemA, HemL, HemB, HemC, and HemD (GenBank: AGX44136.1, AGX44131.1, AGX44132.1, AGX44134.1, AGX44133.4, respectively). For *hemA*, any hit with an E-value lower than 1e-5 was considered *hemA*, and all hits observed were annotated as *hemA*. All *hemL* hits with an E-value less than 1e-100 were considered *hemL*. To be an ALA auxotroph, the genome must be missing both *hemA* and *hemL*. We did not observe any hits to *hemB* with an E-value lower than 1e-5 in any genome predicted to be missing the gene from the annotation-based search. For *hemC*, we required the E-value be less than 1e-29. There were two genomes that had hits below this cutoff. For *hemD*, we required hits to have an E-value less than 1e-40.

Since the *C. saccharobutylicum* HemD is a fusion protein with both the UroIII synthase and UroIII methyltransferases domains, we additionally searched for the *Bacillus subtilis* HemD, which only has the UroIII synthase activity (UniProtKB P21248.2). We did not observe any hits to the *B. subtilis hemD* with an E-value lower than 1e-5 in any genome predicted to be missing the gene from the annotation-based search. Genes with high matches for the *C. saccharobutylicum hemD* were inspected in the IMG browser for domains to determine if the methyltransferase and UroIII synthase domains were both present.

Growth conditions

Clostridium scindens ATCC35704 was grown at 37ºC under 80% N_2, 20% CO_2 in an anaerobic defined mineral salts medium with the following composition (g/L): NaCl, 1; $MgCl_2 \cdot 6H_2O$, 0.5; KH_2PO_4, 0.2; NH_4Cl, 0.3; KCl, 0.3; $CaCl_2 \cdot 2\ H_2O$, 0.015. In addition, 2.29 g of N-Tris(hydroxymethyl)methyl-2-aminoethanesulfonic acid (TES, free acid), 2 ml of a trace element solution (He *et al.*, 2007), 1 ml of a Na_2SeO_3-Na_2WO_4 solution (Widdel and Bak, 1992),10 mg of resazurin, and 40 mg each of the amino acids arginine, cysteine, glycine, histidine, isoleucine, leucine, phenylalanine, proline, serine, threonine, tryptophan, tyrosine, and valine were added per liter (Lovitt *et al.*, 1987). After the medium was boiled and cooled under N_2, the gas was switched to an 80% N_2, 20% CO_2 mix, and the reductants $Na_2S \cdot 9\ H_2O$ and L-cysteine were added to final concentrations of 0.2 mM each. Next, 2.52 g $NaHCO_3$ (30 mM final concentration) was added to the medium, and the pH was adjusted to 7.0. The medium was dispensed under 80% N_2, 20% CO_2 in 10 ml aliquots in 25 ml Balch tubes, or for large volumes, 1 L in 2 L pyrex bottles. Tubes and bottles were sealed with butyl stoppers and aluminum crimp seals, autoclaved for 30 min, and cooled to room temperature. Glucose was subsequently added to a final concentration of 25 mM, and Wolin vitamin solution (Wolin *et al.*, 1963) (prepared

without cobalamin) was added to a final concentration of 1% (v/v). Where indicated, 1 mM ALA was added after autoclaving. The trace element stock solution contained (g/L) Nitriloacetic acid, 1.11; $MnSO_4 \cdot H_2O$, 0.5; $FeSO_4 \cdot 7H_2O$, 0.1; $CoCl_2 \cdot 6H_2O$, 0.1; $ZnCl_2$, 0.1, $NiCl_2 \cdot 6H_2O$, 0.05; $CuSO_4 \cdot 5H_2O$, 0.01; $AlK(SO_4)_2 \cdot 12\ H_2O$, 0.01; H_3BO_3, 0.01; $Na_2MoO_4 \cdot 2H_2O$, 0.01. The $Na_2SeO_3 \cdot Na_2WO_4$ solution contained (g/L): $Na_2SeO_3 \cdot 5H_2O$, 0.006; $Na_2WO_4 \cdot 2H_2O$, 0.008; NaOH, 0.5.

Clostridium sporogenes ATCC 15579 was grown in the same medium and conditions as *C. scindens* with the following changes: cysteine, serine, and threonine were omitted, and 1 mL of a vitamin solution containing 500 mg/L nicotinic acid, 50 mg/L thiamine HCl, 5 mg/L biotin, and 5 mg/L p-aminobenzoic acid was added per liter of medium (Lovitt *et al.*, 1987).

Treponema primitia ZAS-2 was grown at room temperature in anaerobic 4YACo medium with a headspace of 80% H_2, 20% CO_2 as previously described (Graber and Breznak, 2004), with the following changes. For cobalamin added cultures, the final concentration of cyanocobalamin was reduced from 4.42 µM to 37 nM. For testing no addition and ALA addition to cultures, cobalamin-supplemented cultures were serially passaged three times in cobalamin-free medium or in cobalamin-free medium containing 1 mM ALA before being used as inocula for growth experiments. Growth was monitored spectrophotometrically ($O.D._{650}$). All growth experiments were performed in triplicate.

Desulfotomaculum reducens MI-1 was grown anaerobically at 37°C under an N_2 atmosphere in a modified Widdel low phosphate medium with the following per liter: NH_4Cl, 0.25 g; $CaCl_2 \cdot 2\ H_2O$, 0.1 g; $MgCl_2 \cdot 6\ H_2O$, 0.5 g; NaCl, 5 g; KCl, 0.5 g; KH_2PO_4, 0.03 g; TES, 2.292 g; yeast extract, 0.5 g; Na_2SO_4, 2.84 g; $NaHCO_3$, 2.52 g; cysteine-sulfide solution (2.5%), 4 ml; Se/WO solution, 1 ml (Widdel and Bak, 1992); Trace Elements Solution SL-10, 1 ml (Costa *et al.*, 2012); 1000X Wolin's Vitamin solution (without cobalamin), 2 ml. Na-lactate was added as the electron donor (20 mM) and the pH of the medium was adjusted to 7.3 with KOH.

Listeria monocytogenes was grown anaerobically at 25°C under an N_2 atmosphere in medium containing the following per liter: NaCl, 1 g; $MgCl_2 \cdot 6\ H_2O$, 0.5 g; KH_2PO_4, 0.2 g; NH_4Cl, 0.3 g; KCl, 0.3 g; $CaCl_2 \cdot 2\ H_2O$, 15 mg; Na-pyruvate, 2.2 g; yeast extract, 1 g; 1,2-propanediol, 5.9 ml; Trace Elements Solution SL-10, 1 ml; 1000X Wolin's Vitamin solution (without cobalamin), 1 ml; Se/WO solution, 1 ml. The pH of the medium was adjusted to 7.

Blautia hydrogenotrophica DSM 10507 was grown anaerobically in Anaerobic Basal Broth (Hi-Media M1636) under 5% CO_2, 10% H_2, 85% N_2 headspace at 37ºC.

Clostridium kluyveri DSM 555 was grown anaerobically at 33°C under an atmosphere of 80% N_2, 20% CO_2 in medium (pH 6.8-7) with the following per liter: Potassium acetate, 10 g; K_2HPO_4, 0.31 g; KH_2PO_4, 0.23 g; NH_4Cl, 0.25 g; $MgSO_4 \cdot 7\ H_2O$, 0.2 g; yeast extract, 1 g; ethanol, 20 ml; $NaHCO_3$ (9.1% solution), 27 ml; cysteine-sulfide solution (2.5%), 4 ml; Se/WO solution, 0.5 ml; Trace Elements Solution SL-10, 1ml; 1000X Wolin's Vitamin solution (without cobalamin), 1 ml.

Clostridium phytofermentans ISDg (ATCC 700394) was grown anaerobically at 25°C under an N_2 atmosphere in GS-2CB medium (Warnick *et al.*, 2002).

References

Altschul SF, Madden TL, Schäffer AA, Zhang J, Zhang Z, Miller W, *et al.* (1997). Gapped BLAST and PSI-BLAST: A new generation of protein database search programs. *Nucleic Acids Res* **25**: 3389–3402.

Benson DA, Cavanaugh M, Clark K, Karsch-Mizrachi I, Lipman DJ, Ostell J, *et al.* (2013). GenBank. *Nucleic Acids Res* **41**: 36–42.

Costa JC, Barbosa SG, Alves MM, Sousa DZ. (2012). Thermochemical pre- and biological co-treatments to improve hydrolysis and methane production from poultry litter. *Bioresour Technol* **111**: 141–147.

Eddy, SR. (2015). HMMER: biosequence analysis using profile hidden Markov models (v3.1b2) [software]. Available from http://hmmer.org/

Graber JR, Breznak JA. (2004). Physiology and Nutrition of Treponema primitia, an H2/CO2-Acetogenic Spirochete from Termite Hindguts. *Appl Environ Microbiol* **70**: 1307–1314.

Haft DR, Haft DH. (2017). A comprehensive software suite for protein family construction and functional site prediction. *PLoS One* **12**: e0171758.

Hazra AB, Han AW, Mehta AP, Mok KC, Osadchiy V, Begley TP, *et al.* (2015). Anaerobic biosynthesis of the lower ligand of vitamin B12. *Proc Natl Acad Sci* **112**: 10792–7.

He J, Holmes VF, Lee PKH, Alvarez-Cohen L. (2007). Influence of vitamin B12 and cocultures on the growth of Dehalococcoides isolates in defined medium. *Appl Environ Microbiol* **73**: 2847–2853.

Lovitt RW, Morris JG, Kell DB. (1987). The growth and nutrition of Clostridium sporogenes NCIB 8053 in defined media R. *J Appl Bacteriol* 71–80.

O'Leary NA, Wright MW, Brister JR, Ciufo S, Haddad D, McVeigh R, *et al.* (2016). Reference sequence (RefSeq) database at NCBI: Current status, taxonomic expansion, and functional annotation. *Nucleic Acids Res* **44**: D733–D745.

Warnick TA, Methé BA, Leschine SB. (2002). Clostridium phytofermentans sp. nov., a cellulolytic mesophile from forest soil. *Int J Syst Evol Microbiol* **52**: 1155–1160.

Waterhouse AM, Procter JB, Martin DMA, Clamp M, Barton GJ. (2009). Jalview Version 2-A multiple sequence alignment editor and analysis workbench. *Bioinformatics* **25**: 1189–1191.

Widdel F, Bak F. (1992). Gram-negative mesophilic sulfate-reducing bacteria. In: *The Prokaryotes, vol 4*. pp 3352–3378.

Wolin EA, Wolin MJ, Wolfe RS. (1963). Formation of methane by Bacterial Extracts. *J Biol Chem* **238**: 2882–2886.

Supplementary Tables

Supplementary Table 1: All genomes analyzed in this study. Sheet 1 contains the metadata: JGI/IMG identifier, sequencing status, taxonomic data, habitat/ecosystem data, GOLD sequencing project data, genome size, gene count, and number of scaffolds. Sheet 2 is the "genomes vs function" and "genomes vs genes" downloads for the cobamide-dependent enzyme families, cobamide-independent alternatives, and cobamide biosynthesis genes. The number indicates the number of genes that matched the annotation or query gene in that genome. Sheet 3 contains some downloads from a later date that are listed as "#N/A" in sheet 2. Some genome

names had been changed and could not be easily matched to the prior download, as the tool output did not include the unique identifier until recently. Sheet 4 is the “genomes vs functions” download for the 55 single copy genes.

Supplementary Table 2: Genomes in the filtered data set. Sheet 1 contains the metadata, Sheet 2 is the “genomes vs function” and “genomes vs genes” downloads for the cobamide-dependent enzyme families, cobamide-independent alternatives, and cobamide biosynthesis genes. Sheet 3 is the “genomes vs functions” download for the 55 single copy genes.

Supplementary Table 3: Completeness analysis by single copy genes for all genomes. It lists the number of unique single copy genes (maximum 55), the average number of single copy genes, and lists with the annotations either missing or in multiples for each genome.

Supplementary Table 4: Annotations used for cobamide-dependent enzyme families and alternatives and query genes used for BLASTP-based search for enzyme families without annotations.

Supplementary Table 5: Results for cobamide-dependent and –independent enzyme families, and cobamide biosynthesis genes and categories for the filtered data set by genome.

Supplementary Table 6: Strains tested for experimental production of corrinoids in Table 1 and the strain used for genome analysis, the cobamide biosynthesis phenotype, and reference for the corrinoid production observation.

Supplementary Table 7: Sheet 1 contains the definitions of the cobamide biosynthesis pathway sections used in classifying genomes. Sheet 2 contains the definitions of cobamide biosynthesis categories.

Supplementary Table 8: Sheet 1 summarizes the results of the hmmsearch for *bzaABCDEF*. Sheet 2 and 3 show the tabular output from hmmsearch above the trusted cutoff for each HMM for *bzaABDEF* and *bzaC,* which was a domain model, for genomes from GenBank and RefSeq, respectively.

Supplementary Table 9: Genomes with identified tandem CobT homologs based on sequential gene identifiers, and analysis of length of genes. TRUE in column 2 indicates that the tandem annotations were consistent with full-length CobT. Each gene’s JGI/IMG unique identifier, and amino acid length of each gene is listed.

Supplementary Table 10: List of tetrapyrrole precursor salvagers (TPS) checked for missing genes by BLASTP. Genomes in red were excluded as TPS because missing genes completing the tetrapyrrole precursor biosynthesis section were found. The columns for each tetrapyrrole precursor biosynthesis step are populated with results from the search on IMG using annotations. Columns with the BLASTP hits show the bit score and E-value for the highest scoring hit in the genome if found. Results are summarized in the column “Additional genes found by BLAST.” If the genome was consistent with TPS, then the specific type is listed.

Supplementary Figures

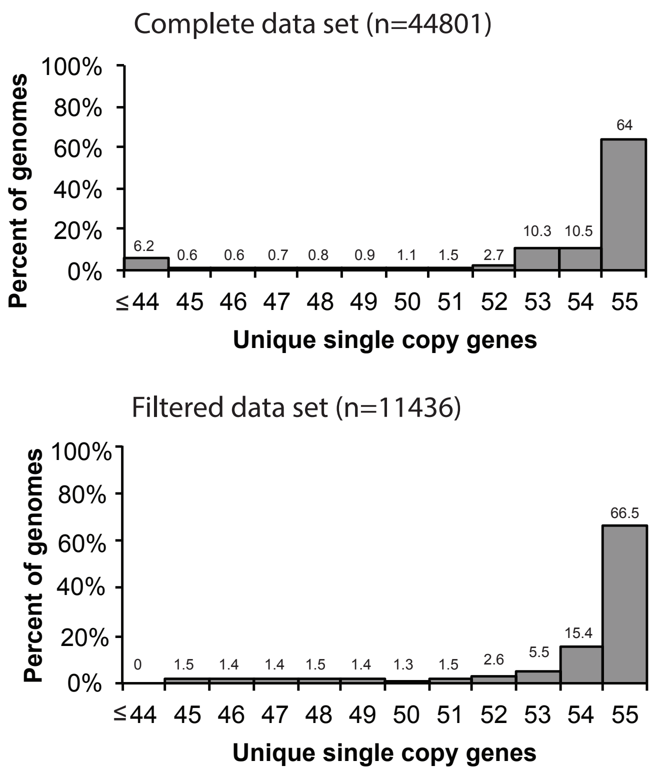

Supplementary Figure 1: Number of unique single copy genes in the bacterial genomes before and after filtering by unique single copy genes and selecting a single genome for each species.

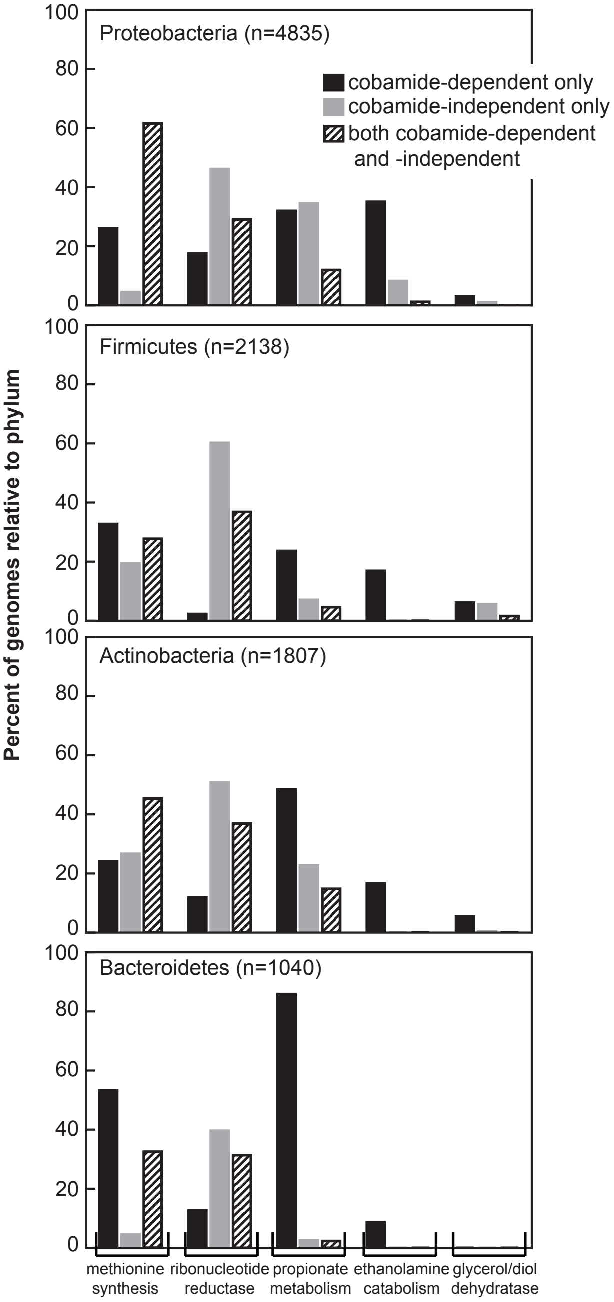

Supplementary Figure 2: Cobamide-dependent enzyme families with cobamide-independent alternatives by phylum.

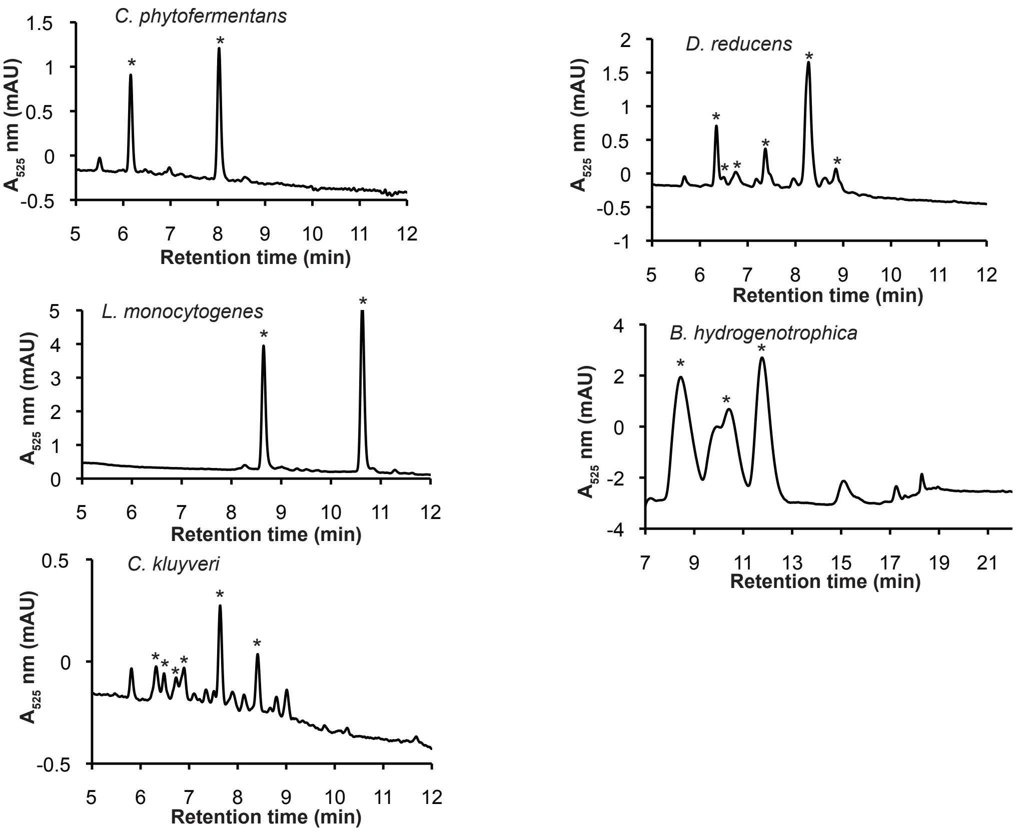

Supplementary Figure 3: Corrinoid extractions from the organisms we tested listed in Table 1 and Supplementary Table 6 were separated on HPLC as described in the methods. Asterisks indicate peaks consistent with a corrinoid based on the UV-Vis spectrum.

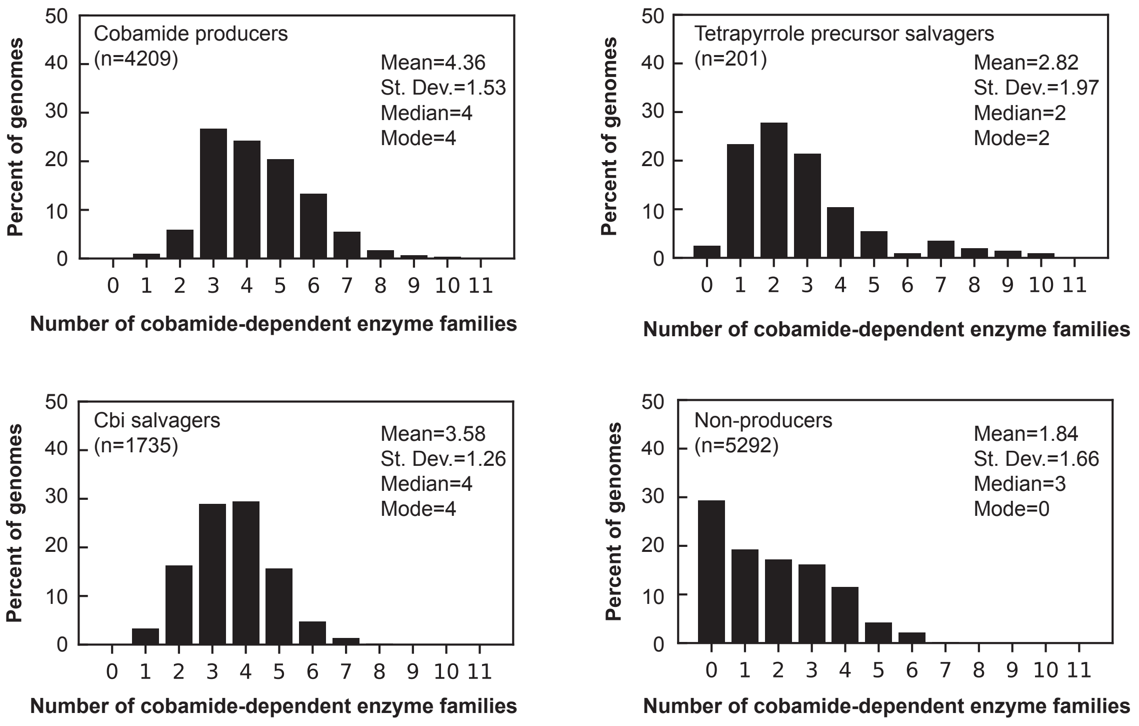

Supplementary Figure 4: Number of cobamide-dependent enzyme families per genome in four cobamide biosynthesis classification categories.
